# PEEK Primary Crowns with Cobalt-Chromium, Zirconia and Galvanic Secondary Crowns with Different Tapers—A Comparison of Retention Forces

**DOI:** 10.3390/ma9030187

**Published:** 2016-03-10

**Authors:** Veronika Stock, Patrick R. Schmidlin, Susanne Merk, Christina Wagner, Malgorzata Roos, Marlis Eichberger, Bogna Stawarczyk

**Affiliations:** 1 Department of Prosthodontics, Dental School, Ludwig-Maximilians-University Munich, Goethestrasse 70, Munich 80336, Germany; stock.veronika@web.de (V.S.); merk.susanne@googlemail.com (S.M.); chrissy.wagner@gmx.net (C.W.); marlis.eichberger@med.uni-muenchen.de (M.E.); 2 Clinic of Preventive Dentistry, Periodontology and Cariology, Center of Dental Medicine, University of Zurich, Plattenstrasse 11, Zurich 8032, Switzerland; patrick.schmidlin@zzm.uzh.ch; 3 Department of Biostatistics, Epidemiology, Biostatistics and Prevention Institute, University of Zurich, Hirschgraben 84, Zurich 8001, Switzerland; mroos@ifspm.uzh.ch

**Keywords:** CAD/CAM, conus crowns, double crowns, electroforming, PEEK, retention force, telescopic crowns

## Abstract

In prosthetic dentistry, double crown systems have proved their suitability as retainers for removable partial dentures. However, investigations in this context, regarding polyetheretherketone, are scarce. Therefore, the aim of this study was to test the retention force (RF) between polyetheretherketone (PEEK) primary and cobalt-chromium (CoCr), zirconia (ZrO_2_) and galvanic (GAL) secondary crowns with three different tapers. Primary PEEK-crowns were milled with the tapers 0°, 1°, and 2° (n = 10/taper, respectively). Afterwards, 90 secondary crowns were fabricated: (i) 30 CoCr-crowns milled from Ceramill Sintron (AmannGirrbach, Koblach, Austria) (n = 10/taper), (ii) 30 ZrO_2_-crowns milled from Ceramill ZI (AmannGirrbach, Koblach, Austria) (n = 10/taper), and (iii) 30 GAL-crowns made using electroforming (n = 10/taper). RF was measured in a pull-off test (20 pull-offs/specimen) and data were analyzed using 2-/1-way Analysis of Variance (ANOVA) followed by the Tukey-Honestly Significant Difference (HSD) post hoc test and linear regression analyses (*p* < 0.05). The measured mean RF values ranged between 9.6 and 38.2 N. With regard to the 0°, 1°, and 2° tapered crowns, no statistically significant differences between CoCr and ZrO_2_ were observed (*p* > 0.141). At 0° taper, no differences in retention forces between GAL, CrCr, and ZrO_2_ crowns were found (*p* = 0.075). However, at 1° and 2° taper, lower RF for GAL-crowns were observed (*p* < 0.009, *p* < 0.001, respectively). According to this laboratory study, PEEK might be a suitable material for primary crowns, regardless of the taper and the material of secondary crown. Long-term results, however, are still necessary.

## 1. Introduction

Polyetheretherketone (PEEK) is a high performance thermoplastic polymer, which consists of an aromatic backbone molecular chain, interconnected by ketone and ether functional groups [1]. Its structure confers outstanding chemical resistance and resistance to thermal and post-irradiation degradation [1]. Its melting temperature is at around 343 °C, and the elastic modulus ranges between 3 and 4 GPa [1]. PEEK presents a lower solubility and water absorption as compared to current esthetic computer-aided-design/computer-aided-manufacturing (CAD/CAM) polymers [2] and is chemically inert [1]. Furthermore, biofilm formation on the surface of PEEK is equal to or even lower than on dental materials, such as titanium and zirconia [3]. Due to these promising physico-mechanical properties, PEEK shows some advantages to traditional alloys and ceramic dental materials.

In prosthetic dentistry, thus far, there has been a versatile use of PEEK for crowns or bridges, clamps in the field of removable dental prostheses, implant supported bars, and provisional abutments [4,5,6,7]. In the field of the double crown technique, studies regarding PEEK are scarce. However, double crown systems containing telescopic crowns with a 0° taper and conus crowns have proved their suitability as retainers for removable partial dentures due to their guidance, support, and protection from dislodging movements [8,9,10,11,12]. They transfer occlusal forces along the long axis of the abutment teeth and are less traumatic than other retainers [8,10,11,12]. Furthermore, double crown dentures can easily be extended if one of the abutment teeth needs extraction [10,12]. These double crown dentures can be designed to be resilient or rigid. Telescopic crowns with a defined occlusal stop and conus crowns are a rigid, non-resilient method, telescopic crowns with no occlusal stop are resilient [8,10].

Cobalt-chromium (CoCr) is very well suited for the double crown technique due to its precise fitting [13], high elastic modulus [14], and mechanical strength [15]. Due to its lower density, CoCr has a lower weight compared to gold alloys [16]. This material presents a high biocompatibility [15] and corrosion resistance [17]. It can be cast or milled from prefabricated homogenous chalky blanks by means of computer-aided-design and computer-aided-manufacturing (CAD/CAM). After milling, the workpieces are sintered to provide the material the final density, size, and mechanical properties [18]. 

Another material also produced by means of CAD/CAM technology is zirconia (ZrO_2_). Due to its high mechanical strength and high biocompatibility [19], ZrO_2_ is used for several medical devices [20]. Not least because of its low surface roughness and its esthetic potential [8], ZrO_2_ has been established in prosthetic dentistry for implants, abutments, different frameworks [19], and as a material for primary crowns in the double crown technique [8]. A very good long-term stability of ZrO_2_ has been confirmed in a 10-year clinical study of fixed dental prostheses with ZrO_2_ frameworks [21].

Uniting the advantages of PEEK with the above-mentioned materials, CoCr and ZrO_2_, is feasible in a double crown technique, *i.e.*, CAD/CAM produced PEEK as primary crown in combination with CAD/CAM-produced CoCr and ZrO_2_ as secondary crowns. In this context, CAD/CAM technology is suggested to be faster and more efficient than conventional methods [18]. Producing secondary crowns using electroforming, as another alternative, also eliminates the hand-modeling phase and fitting [16]. It represents a direct production by electroforming the secondary crown directly onto the primary crown. These galvanic crowns are captivating due to their highly precise fitting. This fitting is essential for proper retentive force and indispensable for normal function of double crown dentures, as mentioned previously [16].

Previous studies measuring the retention force of double crowns examined the influence of different material groups, number of pull-off cycles, and different taper angles [8,16,22,23]. Among double crowns, cylindrical telescopic crowns with parallel walls have to be distinguished from conus crowns with taper angles greater than 0° [11]. Ohkawa and co-workers [23] recommended a maximum taper angle of 2° for long-term use.

This study investigated the influence of CAD/CAM fabricated (milled) CoCr, ZrO_2_, and electroformed GAL secondary crowns on a PEEK primary crown and the influence of three different taper angles, *i.e.*, 0°, 1°, and 2°, respectively. In this context, the retention force was measured and the influence of twenty pull-off cycles was examined. We hypothesized that: (i) different materials of secondary crowns, (ii) taper angles, and (iii) the number of pull-off-cycles have an impact on retention force. 

## 2. Materials and Methods

### 2.1. Fabrication of Primary Crowns

**Figure 1:**
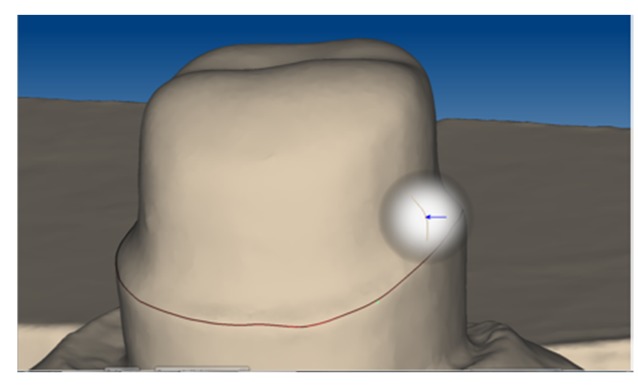
Marking the preparation margin on the scanned abutment tooth.

**Figure 2:**
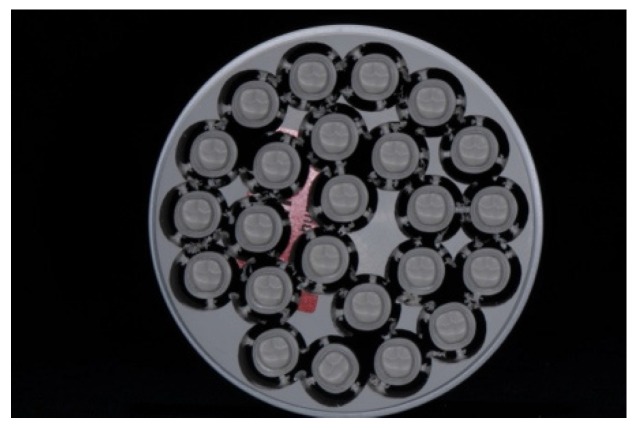
PEEK blank with milled primary crowns of 1° and 2° taper angles.

Thirty metal abutment teeth were cast from a base metal alloy (Remanium GM800+, LOT 936, Dentaurum, Ispringen, Germany,) using an artificial molar tooth as a template for the silicone duplicating technique [22]. The metal abutment teeth were scanned (Ceramill map300, AmannGirrbach, Koblach, Austria; Figure 1) and thirty primary crowns were designed (Ceramill Mind 2.3.0, AmannGirrbach) with three different tapers (0° with a chamfer, 1°, and 2°, n = 10 each). For each taper, 10 primary crowns were milled with CAD/CAM (ZENO Tec System, ZENO 4030 M1, Wieland Dental, Pforzheim, Germany) made of pre-fabricated PEEK blanks (breCAM BioHPP, LOT 394172, bredent, Senden, Germany) (Figure 2). 

After checking the fit of the PEEK primary crowns using Arti-Spray (white, BK 285, Dr. Jean Bausch, Cologne, Germany) and after adaptation on the abutments (where required) using cross cut burs (Komet Dental, LOT 277889, Lemgo, Germany), the crowns were cemented with a self-adhesive resin cement according to the manufacturer’s instruction (RelyX Unicem 2, LOT 509981, 3M ESPE, Seefeld, Germany). To obtain the same path of insertion, samples were fixed in a parallelometer (F4 basic, DeguDent, Hanau, Germany) using pattern resin (Pattern Resin LS, LOT 335201, GC, Tokyo, Japan) and rods corresponding to tapers of 0°, 1°, and 2°. In this way, all samples were embedded in a plaster socket with the path of insertion vertical to the ground (Hera Octastone CN, LOT 3252822, Heraeus Kulzer, Hanau, Germany). A minimal refining of the tapers was processed with an electric high-speed hand piece (W&H Perfecta 900, W&H Dentalwerk Bürmoos, Bürmoos, Austria), which was put in the parallelometer. For this purpose, burs with the respective tapers were used (profile bur tungsten carbide with relief, REF F1372H15 (0°), REF F2002K29 (1°), REF F2002H23 (2°), bredent). All surfaces were finished with silicone polishers (Komet Dental, LOT 307723), polishing brushes (Komet Dental, LOT 226983), and polishing paste (Abraso-Starglanz asg, REF 52000163, bredent) using a handpiece.

### 2.2. Fabrication of Secondary Crowns

In summary, 90 secondary crowns were fabricated consisting of three different materials (Table 1):

1. Thirty milled from CoCr blanks (Ceramill Sintron 71 Blank 16 millimeter, LOT 1303045, AmannGirrbach),
2. Thirty milled from ZrO_2_ blanks (Ceramill ZI 71, LOT 1303002, AmannGirrbach) and
3. Thirty made using electroforming (GAL). All these three groups were fabricated in a non-resilient method with the respective tapers of 0°, 1° und 2° (n = 10 per taper).

**Figure 3:**
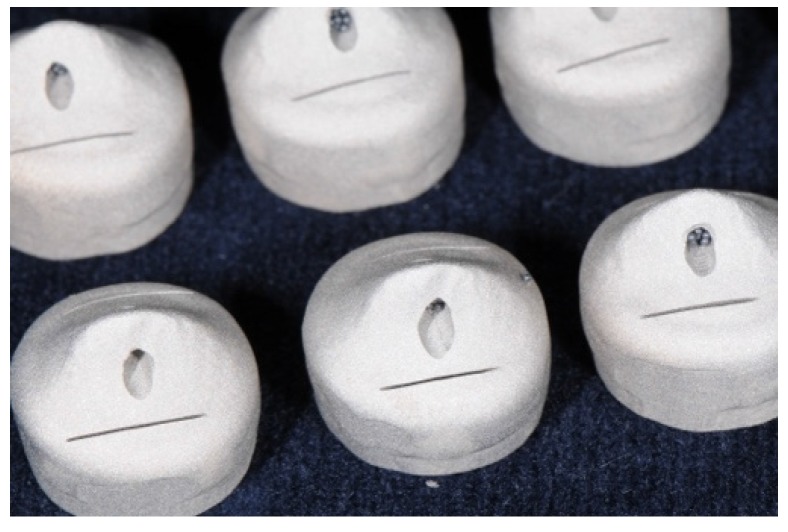
Cobalt-chromium secondary crowns with a hole for the pull-off test directly after sintering.

For the fabrication of the CoCr secondary crowns, each primary crown was scanned (Arti-Spray, white, BK 285, Dr. Jean Bausch; Ceramill map 300, AmannGirrbach) and secondary crowns were designed with a hole for the pull-off test. Then, 30 CoCr crowns were milled of blanks with Ceramill Motion 2 (AmannGirrbach) and the appropriate milling tool (Ceramill Roto Motion 0.6 LOT 20120315; 1.0 LOT 20120605; 2.5 LOT 2010605, AmannGirrbach). For the sintering process, the specimens were set in a sintering furnace (Ceramill Argotherm, AmannGirrbach) at 1 bar pressure of argon and 1.2 bar compressed air, according to the manufacturer’s instruction. The sintered CoCr crowns (Figure 3) were air-abraded using alumina with a mean particle size of 110 µm at 2 bar for 10 s (basic Quattro IS, Renfert, Hilzingen, Germany; Korox 110, LOT 14878430513, Bego, Bremen, Germany). After adaptation on the primary crowns with cross cut burs (Komet Dental, LOT 277889), each secondary crown was polished the same way for 3 min to a high gloss (Abraso-Starglanz asg, REF 52000163, bredent) in order to standardize the technical baseline situation, which might influence the retention force.

**Figure 4:**
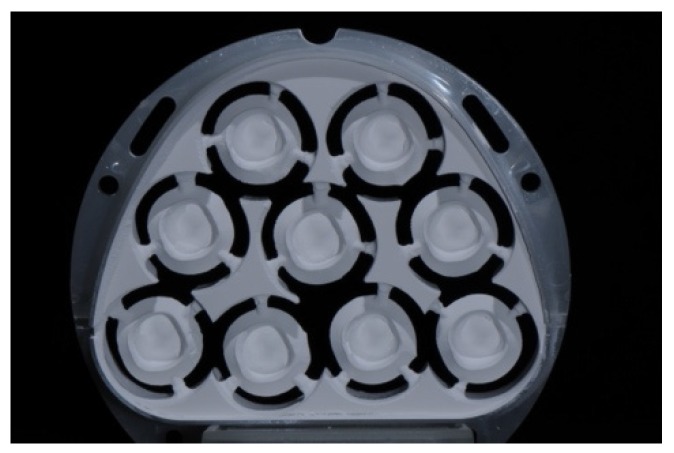
Zirconia blank with milled secondary crowns of 0° taper angle.

For the fabrication of the ZrO_2_ secondary crowns, the PEEK primary crowns were polished, scanned and the secondary crowns were designed with a hole for the pull-off test using the construction parameters for ZrO_2_. Thirty crowns were milled (Ceramill Motion 2; Figure 4) and sintered in a furnace (Ceramill therm, AmannGirrbach) according to the manufacturer’s instructions: A heat-up phase to final temperature of 1450 °C (heating rate 5–10 K/min), 2 h dwell time and a cooling phase to room temperature (5 K/min). Diamond stones and diamond burs (Ceramic Art Set 4371/4369, ZR374M/F, Komet Dental) were used for the whole adaption process. For finishing the inner surface of the ZrO_2_ secondary crowns, each secondary crown was polished the same way for 3 min with a 3-step silicone polishing system (Ceramic Art Set 4371, Komet Dental), round brushes (Komet Dental, REF 9638900190), and polishing paste (YETI DIA-GLACE, Pat 3832085.1, YETI Dentalprodukte, Engen, Germany).

**Figure 5:**
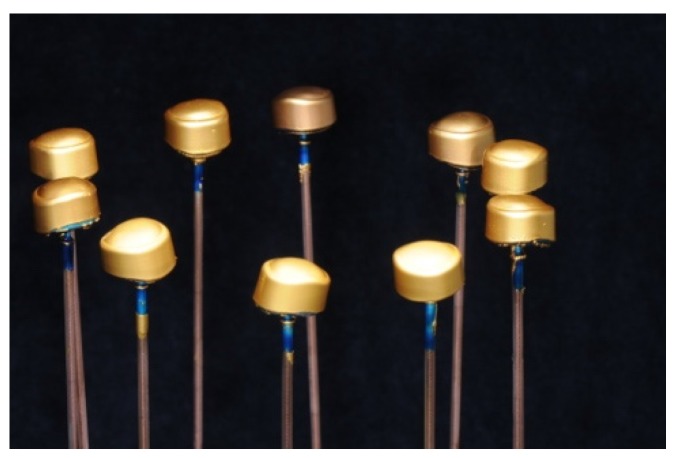
Primary crowns with gold copings on rods after the electroforming process.

The electroforming procedure started with preparing the detached, high gloss polished primary crowns. For the electroforming workflow, a temporary abutment made from a polyurethane resin (Helioform Polyurethane material compound A&B, LOT 512, C. Hafner, Pforzheim, Germany) was necessary. For this purpose, the primary crowns were air-abraded and steam cleaned at the internal side to ensure an adequate adhesion between the inner surface and the polyurethane resin. In the temporary resin abutment, a central borehole was drilled to fix a copper rod using an electroforming anode. After cleaning the primary crowns with ethanol, an airbrush gun was used to apply the silver conductive lacquer (Helioform silver conductive spacer, LOT 02/13, C. Hafner) on the external face of the primary crown according to the manufacturer’s instructions. To link the primary crown with the copper anode, a line of silver conductive lacquer (wide of 2 mm) was manually painted with a brush. To gain a precise galvano formed coping made of gold, light-curing cover lacquer (Helioform cover varnish LC, LOT 122574, C. Hafner) was applied basally and marginally and hardened for 30 s. Fifteen pieces, prepared as described above, were set together on the lid of the galvanic device (Hafner HF Vario Plus, C. Hafner) and plunged in the electrolytic gold bath (Helioform H electrolyte, LOT 00433724, Helioform H concentrate, LOT 0043468, C. Hafner). To achieve the recommended thickness of the gold cope, the program included 255 mA for 14 h (Figure 5). After completion of the electroforming process, the GAL copings (thickness about 0.25 mm) were separated from their primary crowns. Both were cleaned with a 53% solution of nitric acid to solve the silver conductive lacquer. Afterwards, GALs were fixed in a superstructure made of CoCr (AGC Cem Automix system, LOT 697720, Wieland Dental). This was necessary to execute the pull-off tests and to stabilize the delicate structure of GAL. 

### 2.3. Retention Force Measurements

**Figure 6:**
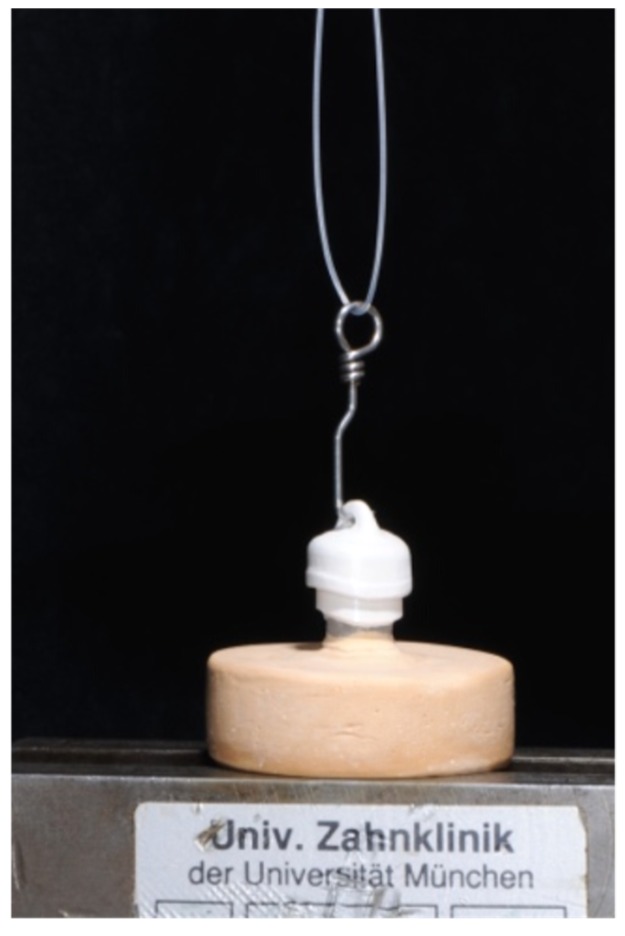
Experimental setup for the pull-off test: a hook pulls a zirconia secondary crown off a PEEK primary crown.

For measuring the retention force the primary crowns were placed in a universal testing machine (Zwick 1445, Zwick, Ulm, Germany) with a 500 N load cell. Each secondary crown was set on their primary crown with artificial saliva as an intermediate layer material (Glandosane, No. 9235461109, cell pharm, Bad Vilbel, Germany). Before each measurement, the power was set to zero and a 50 N weight was set on top of the secondary crown for 20 s [16,24]. The pull-off test was executed by mounting the secondary crown on a hook and pulling them apart using an upper chain. This, together with the path of insertion vertical to the ground, ensured a self-aligning of the complete system. The crosshead speed was set at 50 mm/min [22] and twenty cycles of each specimen were measured (Figure 6). 

### 2.4. Statistics 

A Kolmogorov-Smirnov test was used to verify the normality of data distribution of all groups. In order to analyze the association of pull-off cycles and the retention force, linear regression was applied in each test group. Descriptive statistics were also computed. Significant differences between the groups were verified using 2-way (taper type and secondary crown group) and 1-way ANOVA, followed by the Tukey-HSD post hoc test. All statistical tests were performed with IBM SPSS (Version 20; IBM Corporation, Armonk, NY, USA). Differences were considered statistically significant when *p* was <0.05.

## 3. Results

According to the Kolmogorov-Smirnov test, no violation of normality assumption in all mean retention force values groups was detected, therefore, the data were analyzed using parametric tests. The global results of the descriptive statistics are presented in Table 2. 

The 2-way ANOVA interaction between the taper type and secondary crown group was highly significant (F = 9.857, *p* < 0.001). Therefore, the fixed effects could not be directly compared. Consequently, a 1-way ANOVA, with respect to the hypothesis, was computed. 

Within the crowns with a taper of 0°, no differences in retention forces between GAL, CrCr and ZrO_2_ crowns were found (F = 2.851, *p* = 0.075). Among specimens with 1° and 2° tapers, differences in retention forces between material groups were found (F = 7.911, *p* = 0.002; F = 21.956, *p* < 0.001). According to the Tukey-HSD post hoc test, GAL crowns displayed significantly lower values (9.6 ± 9.08 N; 14.8 ± 8.00 N) than CrCo (21.40 ± 8.11 N; 31.10 ± 11.27 N) and ZrO_2_ (22.80 ± 7.15 N; 38.20 ± 2.39 N) ones (*p* < 0.009; *p* < 0.001). No differences between CoCr and ZrO_2_ crowns in each taper were found (*p* > 0.141) using the Tukey-HSD post hoc test.

Within CoCr crowns, differences between tapers were found (F = 6.214, *p* = 0.006). A taper of 0° (15.00 ± 11.16 N) showed significantly lower retention forces than a 2° (31.10 ± 11.27 N) taper (*p* = 0.004) by means of the Tukey-HSD post hoc test. Within ZrO_2_, 0° (16.90 ± 4.15 N) displayed lower values than the 1° taper (22.80 ± 7.15 N), and 1° lower than the 2° (38.20 ± 2.39 N) taper (F = 49.024, *p* < 0.001, Tukey-HSD post hoc *p* < 0.034). For GAL (F = 5.683, *p* = 0.009), 0° (26.10 ± 15.14 N) tapered crowns showed significantly higher retention force values than those with a 1° (9.60 ± 9.08 N) taper (Tukey-HSD post hoc *p* = 0.007).

According to the linear regression analysis, a significant decrease of the retention force in the CoCr group with 1° taper angle after twenty separation cycles was found (F = 7.341, *p* = 0.007, R^2^ = 0.036; Table 2). The remaining groups showed no impact of pull-off cycles (F < 3.840, *p* > 0.052, R^2^ < 0.020).

## 4. Discussion

For patients wearing a removable partial denture, retention plays a pivotal role [8]. In this study, the influence of material group, taper angle, and number of pull-off cycles on the retention forces were examined. All of them showed a significant impact on retention force values.

Regarding the first hypothesis, relating to the different material groups, the results showed that the GAL crowns behaved differently, compared to CoCr and ZrO_2_: GAL showed significantly lower retention force values than CoCr and ZrO_2_ in 1° and 2° taper groups. This difference can be explained by the production process: The direct production of GAL crowns by the electroforming process ensured an optimal fitting and needed no manual post-processing [16]. For the processing of CoCr and ZrO_2_, in contrast, chalky blanks were milled under dry conditions and sintered first. Afterwards, they required a retention force adjustment by hand, which implied some less predictable retention force values as compared to the GAL crowns [24]. Former studies investigated the difference in retention force between electroformed double crown systems and other materials, but with contrasting results: Bayer *et al.* [25] found that electroformed galvanic crowns showed a higher retention force, whereas Engels *et al.* [24] also found that electroformed crowns showed a lower retention force compared to cast ones. Furthermore, CoCr and ZrO_2_ mainly adhere through friction and wedging, whereas galvanic crowns basically adhere by hydraulic adhesion. It can be assumed that the variation in the results is not only material related, but also especially related to the manufacturing techniques. Sandblasting CoCr and ZrO_2_ influences surfaces roughness. The roughened surface, friction, and wedging could be an explanation for the higher retention force values of CoCr and ZrO_2_ as compared to GAL. Due to the fact that CoCr and ZrO_2_ were manufactured using a milling system, and were not cast with a refractory investment material, may lead to the question of why the surfaces were sandblasted and why roughening was introduced. The reason for selecting approach was simply that CoCr crowns should be sandblasted according to the manufacturer`s instruction of the Ceramill Argotherm oven for Ceramill Sintron. This aspect, however, must be kept in mind when interpreting the results of our study. Furthermore, the viscosity of the applied saliva, as well as the chamfer design, may influence the hydraulic adhesion and, thus, the retention force as well [8,26]. 

The second hypothesis showed that, among all three material groups, the taper angle had an impact on retention force values. CoCr and ZrO_2_ secondary crowns with a taper of 2° showed higher retention force values than crowns with 0°. However, this fact contradicts previous studies [11,23], which showed a decrease in retention force when the taper angle increased. CoCr and ZrO_2_ both present a high elastic modulus (200 GPa) and are rigid and stable (280 HV 10; 1200 HV 10) [14,19]. PEEK, in contrast, is soft and ductile (110 HV 5/20) and shows a low elastic modulus (4 GPa). Putting a CoCr or ZrO_2_ secondary crown on a PEEK primary crown could lead to a strong wedging due to the flexibility of PEEK and the differences in elastic modulus. This could be a possible explanation as to why 2° tapered crowns show higher retention force values than 0° tapered crowns.

In contrast, GAL secondary crowns with a 0° taper angle showed higher retention force values than 1° tapered crowns, as expected. This behavior corresponds to findings of previous studies mentioned above [11,23], which showed that an increase in taper angle leads to a decrease of retention force. Galvanic copings with high ductility and relatively low elastic modulus (approximate 80 GPa) have similar properties to PEEK. Güngör *et al.* [11] and Ohkawa *et al.* [23] used a gold-silver-palladium alloy both for primary and secondary crowns, which also means identical mechanical properties. Therefore, their result of increasing retention force with decreasing taper may only be right when both crowns have comparable properties. Another reason for the higher retention force of 0° tapered galvanic crowns could be the chamfer. The latter represents a sealing between primary and secondary crown, which is necessary for creating hydraulic adhesion [8]. 

The linear regression showed a significant impact of twenty pull-off cycles on retention force values. This implies that the third hypothesis showed a significant decrease of retention force values in the group of cobalt-chromium with a 1° taper angle. However, this was the only group of the nine that decreased after twenty pull-off cycles. Due to the fact that the other eight groups showed stable retention force values, it can be assumed that PEEK might be a suitable material for primary crowns, regardless of the taper and the material of the secondary crown. A potential explanation could again be that PEEK is a soft and ductile material that yields and adapts well. The low elastic modulus and the ductility are reasons for the good processability of PEEK. The adaption process appeared easy, which resulted in a good marginal fit. In contrast, polishing appeared difficult but was necessary for an optimal running surface and subsequent measurements. However, these measurements must be seen as a basic initial testing of the PEEK material in combination with approved materials, such as CoCr, ZrO_2_, and GAL crowns.

Another shortcoming of this investigation is the fact that the tested specimens were not exposed to thermo-mechanical stress, which occurs under daily wear. Nevertheless, the fact that stable retention force values were measured after twenty pull-off cycles suggests a good forecast regarding long-term investigations. GAL crowns might show less wear, as there is no direct contact between the primary and secondary crown [8].

Altogether, satisfactory high retention force values were achieved, which shows that PEEK, in combination with cobalt-chromium, zirconia, as well as with galvanic secondary crowns, is suitable as a primary crown for removable partial dentures. In the 1° and 2° tapers, CoCr and ZrO_2_ presented higher retention force values than GAL, whereas in the 0° taper no difference was found.

## Figures and Tables

**Table 1:** An overview of all materials, product names and fabrication methods of primary and secondary crowns.

	Primary Crown	Secondary Crown	Secondary Crown	Secondary Crown
Material	PEEK	CoCr	ZrO2	GAL
Product name	BioHPP blank	Ceramill Sintron blank	Ceramill ZI blank	Helioform H electrolyte/concentrate
Method	Milled	Milled and sintered	Milled and sintered	Electroformed

**Table 2:** Mean retention force values (N), one-way ANOVA results and slopes with corresponding F/P and R^2^ values according to linear regression for all tested groups.

Groups	Mean ± SD	Slope	F-Values	P-Values	R^2^
0° taper angle					
CoCr	15.00 ± 11.16 ^a/A^	–0.249	3.384	0.067	0.017
ZrO_2_	16.90 ± 4.15 ^a/A^	–0.080	2.438	0.120	0.012
GAL	26.10 ± 15.14 ^b/A^	–0.309	2.963	0.087	0.015
1° taper angle					
CoCr	21.40 ± 8.11 ^ab/B^	–0.267	7.341	0.007	0.036
ZrO_2_	22.80 ± 7.15 ^b/B^	–0.177	3.831	0.052	0.019
GAL	9.60 ± 9.08 ^a/A^	–0.022	0.040	0.842	0.001
2° taper angle					
CoCr	31.10 ± 11.27 ^b/B^	–0.155	1.105	0.294	0.006
ZrO_2_	38.20 ± 2.39 ^c/B^	–0.056	1.259	0.263	0.006
GAL	14.80 ± 8.00 ^ab/A^	–0.086	0.536	0.465	0.003

^a,b^ differences between different tapered crowns within one material group; ^A,B^ differences between the material groups within one taper type.
